# Special Issue on the “Regulation and Physiopathology of the Gut Barrier”

**DOI:** 10.3390/ijms231810638

**Published:** 2022-09-13

**Authors:** Sophie Thenet, Véronique Carrière

**Affiliations:** 1 Sorbonne Université, INSERM, Centre de Recherche Saint-Antoine, CRSA, F-75012 Paris, France; 2 Paris Center for Microbiome Medicine (PaCeMM) FHU, APHP, F-75012 Paris, France; 3 EPHE, PSL University, F-75014 Paris, France

The importance of gut barrier integrity in intestinal homeostasis and the consequences of its alteration in the etiology of human pathologies have been subjects of exponentially growing interest during the last decade. The gut barrier is a complex functional unit in which several actors cooperate through direct or indirect interactions. This Special Issue, “Regulation and Physiology of the Gut Barrier”, was an opportunity to highlight emerging and overlooked aspects of the physiological regulation of the intestinal barrier function in addition to their links with various pathologies, including new findings on potential therapeutic strategies. In this editorial, the original articles and reviews of this Special Issue are briefly presented in the general context of the current knowledge on gut barrier function (Figure 1).

## 1. Cooperation of Different Actors of the Intestinal Barrier during Gut Homeostasis

The gut barrier function encompasses (i) actors in the luminal compartment hosting the microbiota separated from the mucosa by a mucus layer, (ii) a highly polarized epithelial monolayer, which controls paracellular permeability through the integrity of cell–cell junctions (especially tight junctions), and (iii) the lamina propria as well as the gut-associated lymphoid tissue (GALT), which harbors a large number and variety of immune cells.

In the intestinal lumen, commensal bacteria and a mucus layer both contribute to the integrity of the gut barrier by establishing protection against pathogen invasion and by educating immune cells [8]. Indeed, studies in germ-free animals revealed that the absence of microbiota is associated with a thinner mucus layer [9,10], which can be restored by providing microbiota-derived products such as lipopolysaccharides (LPS) or peptidoglycans [9]. Reciprocally, mice deficient in the expression of Muc2, the main mucin composing the intestinal mucus layer, spontaneously display bacterial overgrowth and susceptibility to inflammation, with some characteristics close to those of human ulcerative colitis [11,12]. Intricate interactions between mucus and microbiota were reported. Several bacterial species are known to promote the synthesis of mucus or its degradation [13]. Changes in microbiota composition may also influence mucus properties through the modification of mucin glycosylation [13]. Thanks to the mucus layer, along with the luminal release of secreted IgA arising from plasma cells in the lamina propria and transcytosis, direct interactions between commensal bacteria and the intestinal epithelium do not take place in physiological conditions, with the exception of segmented filamentous bacteria (SFB), which play a crucial role in the education of the intestinal immune system [14]. Furthermore, gut microbiota exert a profound influence on epithelial cells’ properties through the production of metabolites such as bile acids, short-chain fatty acids, tryptophan-metabolism-derived products, and quorum sensing molecules [15,16,17,18].

The intestinal epithelium located at the interface between the intestinal lumen and the lamina propria is a monolayer composed of several cell types [19,20]. The enterocytes (in the small intestine) or colonocytes (in the colon) represent about 90% of the cells of the epithelium cell monolayer. These cells are involved in nutrient absorption and antimicrobial defense. Goblet cells, representing about 20% of intestinal epithelial cells, with a higher proportion in the colon, are responsible for the secretion of mucus into the intestinal lumen. Enteroendocrine cells represent 1% of the intestinal epithelial cells and are sparsely dispersed along the intestine. They secrete several enterohormones, such as glucagon-like peptide-1 (GLP-1) and GLP-2, which are involved in epithelium homeostasis and energetic metabolism. In this Special Issue, Osinski et al. [1] provide an update on the knowledge of the enteroendocrine system and its impact on the function of other organs. The authors stress that little is known about the impact of enterohormones on gut barrier function; however, they highlight a link between GLP-2 and the integrity of the intestinal epithelial barrier. Other differentiated cell types, such as tuft cells, Paneth cells, and M-cells, contribute to gut homeostasis and host defense by secreting the parasite-induced cytokine IL-25, antimicrobial peptides, and by presenting bacterial or dietary antigens to immune cells, respectively [21].

The intestinal epithelium undergoes a constant and rapid renewal through coordinated cell proliferation, migration, differentiation, and extrusion processes [22]. During this highly dynamic turnover, the integrity of the epithelial barrier must be maintained. The shedding of intestinal epithelial cells is one of the critical points, as its disturbance can lead to vulnerable penetrable sites, disrupting the barrier. Here, Ngo et al. [2] detail the various mechanisms involved in cell extrusion and cell death, which differ during gut epithelium physiological turnover and in pathological conditions such as inflammation and cancer. In particular, the authors underline the importance of cell–cell junction remodeling during these processes.

Cell–cell junctions, and tight junctions (TJs) in particular, are essential in maintaining the integrity of the intestinal epithelial barrier. TJs control the paracellular flux of ions and molecules on either side of the epithelium with both size and charge selectivity [23,24]. Two distinct paracellular fluxes, called “pore” and “leak” pathways, have been defined [25]. The pore pathway refers to a high-capacity flux depending on the sizes and charges of molecules, whereas the leak pathway is a low-capacity route with limited selectivity. The review of Monaco et al. [3] in this Special Issue recapitulates the characteristics of the leak pathway, which remains less understood and still more debated than the pore pathway. As a dysregulated passage of macromolecules through the intestinal epithelium has distinct pathophysiological consequences from the disturbance of ionic fluxes, a thorough knowledge of this specific paracellular pathway is required to determine if it could be specifically targeted in therapeutic strategies.

At the interface between the intestinal lumen and circulation, the intestinal epithelial cell monolayer is sensitive to signals from both compartments and mediates the crosstalk between gut microbes and host immunity [21,26]. Intestinal epithelial cells participate in the fine-tuning of the immune response to enable efficient defense against pathogens while maintaining tolerance to innocuous stimuli [24,26]. Innate and adaptive immunity recognition receptors expressed by epithelial cells are responsive to microbe-associated molecular patterns (MAMPs) and mediate this dialogue [27]. The intestinal immune system is composed of immune cells located either within the epithelium, dispersed in the lamina propria, or organized in sub-epithelial structures called gut-associated lymphoid tissue (GALT). As a whole, it harbors a large variety of innate and adaptive immune cells, such as dendritic cells, macrophages, natural killer cells, and lymphocytes [28]. In addition to “professional” immune cells, intestinal epithelial cells are also able to secrete chemokines such as interleukin-8 (in response to MAMPs or to epithelial barrier leakage), which in turn leads to the recruitment and/or differentiation of immune cells [29,30]. The maturation of the intestinal immune system is dependent on the microbiota. Indeed, the absence of commensal bacteria in germ-free animals is associated with important defects in intestinal lymphoid nodes’ architectures and functions [8]. Moreover, by promoting the production via intestinal epithelial cells of several factors, such as transforming growth factor beta (TGF-β) or indoleamine 2,3-dioxygenase, the colonization of germ-free mice with *Clostridium* species increased the number of regulatory T lymphocytes [31], which are essential for the tolerance of commensal bacteria and dietary antigens. Recently, quorum sensing molecules produced during bacteria communication have been shown to directly modulate cytokine secretion by immune cells [32]. On the other side, certain cytokines secreted by immune cells contribute to maintaining the integrity of the epithelial barrier and intestinal homeostasis [33,34], whereas others exert deleterious effects (see Section 2).

Recently, Rath and Haller [35] introduced the concept of the “metabolic injury” of epithelial cells as a potential causing factor in the development of intestinal inflammation. This concept places the metabolic changes in epithelial cells, in particular, the modification of oxidative metabolism, at a central position in the dialogue between microbiota and immune cells. Hypoxia- or microbiota-derived metabolites, such as aryl hydrocarbon agonists or short-chain fatty acids known to alter glucose or energy metabolism [36,37,38], can modulate several actors of the barrier, establishing a strong link between epithelial cell metabolism and intestinal barrier function [39,40,41,42].

## 2. Intestinal Barrier Dysfunction in Pathologies

Many pathological conditions, such as graft versus host disease, and accidental radiation exposures or radiotherapy [43,44], as well as several infectious diseases involving bacterial, fungal, or viral pathogens, lead to the disruption of the intestinal barrier integrity, with different degrees of severity. In this Special Issue, Paradis et al. [4] provide an overview of the mechanisms used by pathogens to perturb the intestinal barrier through alterations of tight junction integrity. Direct and indirect mechanisms are involved. Pathogens can directly alter TJ integrity by acting on the actin cytoskeleton, modifying intracellular calcium levels, or changing the expression or the localization of TJ proteins. Pathogens can also indirectly impact TJ integrity through the modulation of the inflammatory response during the invasion.

In addition to the pathogenicity of certain living bacteria, bacterial fragments, such as LPS, also play an important role in many chronic diseases via their ability to activate inflammatory pathways in immune and epithelial cells [45]. The deleterious effects of LPS depend on their nature as a function of bacteria species [46]. An increased passage of microbiota-derived antigens through a damaged intestinal barrier activates immune cells, contributing to intestinal and systemic inflammation, which in turn directly impairs TJ integrity via secreted cytokines and creates a vicious circle.

In several pathologies, such as intestinal bowel disease (IBD), obesity, diabetes, and possibly other metabolic or neurologic disorders, intestinal barrier defects are observed, but their causal role is difficult to establish since they are intricately linked with gut microbiota dysbiosis and with an exacerbated intestinal immune response [47,48,49]. In such pathologies, barrier impairment, including intestinal hyperpermeability, could intervene early in the disease [50,51], and has been proposed as a second gastrointestinal hit after the establishment of dysbiosis in metabolic disease [52]. Studies on mouse models of cell–cell junction modification argue that a mild alteration of the intestinal barrier is not sufficient per se to trigger disease, but rather induces subclinical mucosal immune responses, which sensitize mice to intestinal injuries leading to inflammation [25,53,54]. Therefore, the relevance of targeting actors of the epithelial barrier function, such as the integrity of cell–cell junctions in therapeutic approaches, remains an open question that requires more in-depth knowledge [55].

A direct harmful effect of several proinflammatory cytokines on intestinal epithelial monolayer integrity, and, in particular, on cell–cell junctions, has been demonstrated. The molecular mechanisms involved in the alteration of the intestinal epithelial barrier by the proinflammatory cytokine tumor necrosis factor-α (TNF-α) have been well-described. High levels of TNF-α are found in the intestinal mucosa of patients with IBD or obesity [56,57]. After the binding on its receptor at the surface of intestinal epithelial cells, TNF-α activates a signaling cascade that leads to the stimulation of the nuclear factor-kappa B (NF-κB) pathway, which in turn increases the expression of myosin light-chain kinase (MLCK), an important regulator of the actin–myosin belt at the level of tight junctions [25,58]. The excessive contraction of the actin cytoskeleton via MLCK results in an increase in paracellular permeability [25]. Moreover, TNF-α induces intestinal epithelial cell death through mechanisms involving the NF-κB pathway, protein kinases, and caspase activation [59]. Currently, treatment with anti-TNF-α antibodies or TNF-α inhibitors constitutes an important therapeutic approach in IBD, particularly for patients with Crohn’s disease [60]. However, due to the side effects of long-term treatments with TNF-α inhibitors, new strategies to restore gut barrier function and mitigate inflammation are currently under investigation. Some of them evaluate the effects of existing drugs already used in other contexts to treat several pathologies.

The activation of opioid receptors has gained attention for their anti-inflammatory effects in various diseases, including IBD. It has been observed that immune cells in the periphery can secrete opioid peptides, which act on neurons to decrease nociception. The secretion of opioid peptides can be observed locally at the site of tissue inflammation after the recruitment of immune cells [61]. In this Special Issue, Mas-Orea et al. [5] studied the importance of opioid receptors in the gut barrier and inflammation in a mouse model of colitis. Using an antagonist of peripheral opioid receptors, the authors showed that an endogenous opioid tone contributes to maintaining gut barrier integrity and to controlling the immune response. Contrary to endogenous opioids, exogenous opioids, such as morphine, have detrimental effects on the gut barrier [62]. Targeting endogenous opioid receptor stimulation could represent a promising approach to treating gut barrier dysfunction and inflammation, taking into account the potential dual face of such molecules.

Metformin, widely used in the treatment of diabetes, is known to exert other various effects, including the modulation of the immune response and gut microbiota composition [63,64]. Recent studies have highlighted the protective role of metformin in the gut barrier [65,66] through the activation of adenosine-monophosphate-activated protein kinase (AMPK), a protein known to regulate cell energy metabolism but also tight junction assembly [67]. In this Special Issue, Jang et al. [6] studied the effect of metformin on a mouse model of radiation-induced enteropathy, on gut organoids, and on an epithelial cell culture model. The authors observed that metformin treatment stimulates epithelial cell proliferation, associated with an increased expression of stem cell markers and increased goblet cell number in an irradiated epithelium. Such a treatment alleviates epithelial damage and inflammation in irradiated animals.

Along with pharmacological interventions, nutrient modulation represents a strategy for modulating intestinal barrier function [68,69,70]. Enteral nutrition is currently used to treat young patients suffering from gastrointestinal diseases, particularly Crohn’s disease [71]. Several mechanisms involved in the beneficial effect of enteral nutrition have been proposed [72]. First, the defined composition of delivered nutrients excludes antigens that can activate immune cells and induce inflammation while providing beneficial nutrients. Secondly, changes in microbiota composition have been observed following enteral nutrition, which may modulate dysbiosis and enhance the production of beneficial gut-derived metabolites. The reduction in systemic markers of inflammation observed in patients treated with enteral nutrition may be the result of these two actions. In this Special Issue, Boumessid et al. [7] provide an overview of the use of the Modulen^®^ formula for enteral nutrition. The authors discuss the importance of the composition of this formula, stressing the beneficial effect of its high TGF-β2 content. This cytokine is known to prevent goblet cell depletion, reinforce cell–cell junctions, and to exert an anti-inflammatory effect [73].

Other approaches to restoring gut homeostasis aim at modifying the composition of gut microbiota. Several studies have shown that *Lactobacillus plantarum* or *Lactobacillus rhammnosus* exert protective effects in cell culture models mimicking intestinal epithelial barrier alteration. Multiple mechanisms, including the reinforcement of the expression or localization of TJ proteins at cell–cell junctions in human epithelial cells, have been described [74,75]. Other studies have reported a beneficial effect of *Akkermansia muciniphila* on gut barrier function [76]. The abundance of this bacterium is reduced in patients with IBD or obesity [76]. A protective effect of *A. muciniphila* administration on gut barrier function and mucosal inflammation in a mouse model of colitis was observed [77,78]. Part of the effect occurs through the increase in short-chain fatty acid production, known to participate in the integrity of the epithelial barrier, and through an increased secretion of mucus [76]. However, the clinical efficiency of such probiotic treatments remains modest. Manipulating gut microbiota via a fecal microbiota transplantation represents a promising approach for the treatment of pathologies associated with dysbiosis. Such a treatment provides an important beneficial effect for patients with a *Clostridioides difficile* infection [79,80]. The efficiency of a fecal microbiota transplantation for other pathologies is under evaluation [81]. The standardization of the procedure is still ongoing. Specifically, the characteristics of donors and recipients need to be better defined to improve the efficiency and safety of fecal microbiota transplantations [82,83].

## 3. Concluding Remarks

The intestinal barrier function is controlled by many factors and actors originating (i) from the intestinal lumen, such as microbes, microbiota metabolism-derived metabolites, the mucus layer, or nutrients; (ii) from the epithelial cell monolayer, such as cell–cell junction integrity, the secretion of antimicrobial peptides, or cell metabolism; and (iii) the mucosal adaptive and innate immune cells as well as their secreted mediators, such as secretory IgA and cytokines.

Despite the tremendous number of publications on intestinal barrier regulation in health and diseases during this last decade, this Special Issue shows that numerous fields remain to be explored. In particular, whether and how the intestinal epithelium could represent a direct target for the development of new therapeutic strategies is still an open question. In addition, disentangling the multiple crosstalks between the actors of gut barrier function will take advantage of emerging models, such as human-microbiota-associated mouse models, gut-on-chip, or dynamic simulators of the human digestion system [84], which better recapitulate this fascinating and complex functional unit.

## Figures and Tables

**Figure 1:**
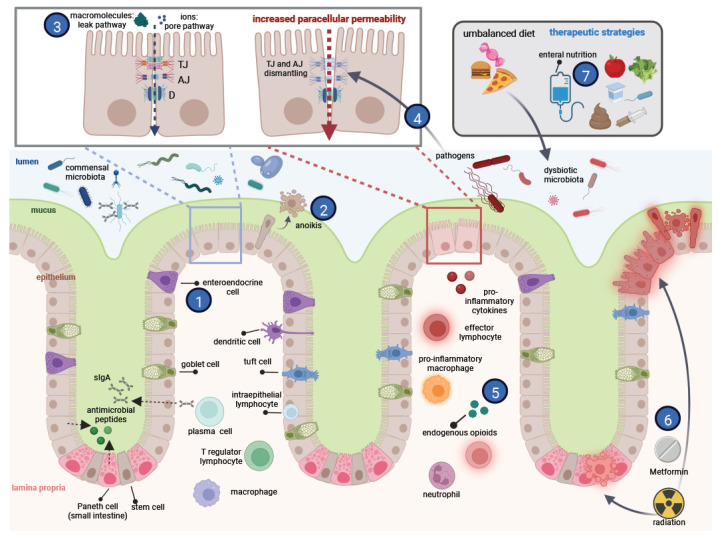
**Actors and regulation of gut barrier function.** The central actor of the intestinal barrier is a single-monolayer epithelium, which undergoes a rapid renewal from stem cells (located at the bottom of crypts) followed by the differentiation of proliferating cells into several specialized cell types. Cells of the absorptive lineage (enterocytes/colonocytes) ensure nutrient absorption, whereas the secretory lineage comprises several cell types involved in other gut functions, including its barrier function: Paneth cells (in the small intestine but not the colon) synthesize antimicrobial peptides, and goblet cells secrete mucins and other proteins that comprise the mucus layer. The role of enteroendocrine cells, secreting a large variety of enteric hormones, in the barrier function is discussed by Osinski et al. [1]. At the villus apex in the small intestine (or at the surface epithelium in the colon), cells are shed in the lumen and die by anoikis. How the barrier integrity is maintained despite constant cell extrusion is discussed by Ngo et al. [2]. A major actor in the epithelial barrier function is the apical junctional complex, consisting of tight junctions (TJs), adherens junctions (AJs), and desmosomes (D). TJs control the paracellular permeability by differentially regulating the flux of ions (pore pathway) or macromolecules (leak pathway described here by Monaco et al. [3]); the deregulation of the latter pathway promotes the passage of potentially harmful molecules, which can trigger exacerbated immune responses. Many TJ proteins, associated cytoskeletons, and signaling pathways are targeted by intestinal pathogens, weakening the barrier function through mechanisms described by Paradis et al. [4]. Gut homeostasis relies on tripartite crosstalk between the epithelial cells, the microbiota, and the intestinal immune system. The gut mucosa harbors innate and adaptive immune cells, which cooperate to tolerate dietary antigens and the commensal microbiota while being capable to eliminate invading pathogens. In inflammatory bowel disease, dysbiosis and activated immune cells secreting proinflammatory cytokines exert harmful effects on epithelial cells and may generate a vicious circle of barrier disruption and inflammation. Endogenous opioid peptides locally secreted by T cells could have beneficial effects on epithelial integrity in this inflammatory environment as shown by Mas-Orea et al. [5]. Dramatic epithelial destruction can occur in the intestine upon accidental radiation or during radiotherapy. Metformin, a widely used anti-diabetic drug, was shown by Jang et al. [6] to protect epithelial barrier function in a mouse model of radiation-induced enteropathy. Gut microbiota are key actors in the intestinal barrier. Commensal species compete with pathogens for intestinal niches and nutrients, and release antibacterial molecules as well as many metabolites that exert beneficial effects on host cells. Imbalanced nutrition and inflammation can lead to dysbiotic microbiota and the loss of beneficial metabolites. Strategies to restore microbiota diversity include a balanced diet, probiotic administration, or a fecal microbiota transplantation. In Crohn’s disease, enteral nutrition has several beneficial effects: it promotes the healing of the mucosa and also modulates the composition of the microbiota, as discussed by Boumessid et al. [7]. AJ: adherens junctions; D: desmosomes; TJ: tight junctions; and sIgA: secretory IgA. Numbers in blue circles correspond to the cited references. Created with BioRender.com (accessed on 30 July 2022).

## Data Availability

Not applicable.

## Abbreviations

AMPK	adenosine monophosphate-activated protein kinase
GALT	gut-associated lymphoid tissue
GLP-1	glucagon-like peptide-1
GLP-2	glucagon-like peptide-2
IBD	intestinal bowel disease
LPS	lipopolysaccharides
MLCK	myosin light-chain kinase
NF-κB	nuclear factor-kappa B
TGF-β	transforming growth factor-beta
TJ	tight junction
TNF-α	Tumor necrosis factor-α

## References

[1] Osinski C., Moret D., Clément K., Serradas P., Ribeiro A. (2022). Enteroendocrine System and Gut Barrier in Metabolic Disorders. Int. J. Mol. Sci..

[2] Ngo P.A., Neurath M.F., López-Posadas R. (2022). Impact of Epithelial Cell Shedding on Intestinal Homeostasis. Int. J. Mol. Sci..

[3] Monaco A., Ovryn B., Axis J., Amsler K. (2021). The Epithelial Cell Leak Pathway. Int. J. Mol. Sci..

[4] Paradis T., Bègue H., Basmaciyan L., Dalle F., Bon F. (2021). Tight Junctions as a Key for Pathogens Invasion in Intestinal Epithelial Cells. Int. J. Mol. Sci..

[5] Mas-Orea X., Sebert M., Benamar M., Petitfils C., Blanpied C., Saoudi A., Deraison C., Barreau F., Cenac N., Dietrich G. (2021). Peripheral Opioid Receptor Blockade Enhances Epithelial Damage in Piroxicam-Accelerated Colitis in IL-10-Deficient Mice. Int. J. Mol. Sci..

[6] Jang H., Kim S., Kim H., Oh S.H., Kwak S.Y., Joo H.-W., Lee S.B., Jang W.I., Park S., Shim S. (2022). Metformin Protects the Intestinal Barrier by Activating Goblet Cell Maturation and Epithelial Proliferation in Radiation-Induced Enteropathy. Int. J. Mol. Sci..

[7] Boumessid K., Barreau F., Mas E. (2021). How Can a Polymeric Formula Induce Remission in Crohn’s Disease Patients?. Int. J. Mol. Sci..

[8] Zheng D., Liwinski T., Elinav E. (2020). Interaction between microbiota and immunity in health and disease. Cell Res..

[9] Petersson J., Schreiber O., Hansson G.C., Gendler S.J., Velcich A., Lundberg J.O., Roos S., Holm L., Phillipson M. (2011). Importance and regulation of the colonic mucus barrier in a mouse model of colitis. Am. J. Physiol.-Gastrointest. Liver Physiol..

[10] Sharma R., Schumacher U., Ronaasen V., Coates M. (1995). Rat intestinal mucosal responses to a microbial flora and different diets. Gut.

[11] Van der Sluis M., De Koning B.A., De Bruijn A.C., Velcich A., Meijerink J.P., Van Goudoever J.B., Büller H.A., Dekker J., Van Seuningen I., Renes I.B. (2006). Muc2-deficient mice spontaneously develop colitis, indicating that MUC2 is critical for colonic protection. Gastroenterology.

[12] Wenzel U.A., Magnusson M.K., Rydström A., Jonstrand C., Hengst J., Johansson M.E.V., Velcich A., Öhman L., Strid H., Sjövall H. (2014). Spontaneous Colitis in Muc2-Deficient Mice Reflects Clinical and Cellular Features of Active Ulcerative Colitis. PLoS ONE.

[13] Paone P., Cani P.D. (2020). Mucus barrier, mucins and gut microbiota: The expected slimy partners?. Gut.

[14] Schnupf P., Gaboriau-Routhiau V., Sansonetti P.J., Cerf-Bensussan N. (2017). Segmented filamentous bacteria, Th17 inducers and helpers in a hostile world. Curr. Opin. Microbiol..

[15] Gasaly N., de Vos P., Hermoso M.A. (2021). Impact of Bacterial Metabolites on Gut Barrier Function and Host Immunity: A Focus on Bacterial Metabolism and Its Relevance for Intestinal Inflammation. Front. Immunol..

[16] Ghosh S., Whitley C.S., Haribabu B., Jala V.R. (2021). Regulation of Intestinal Barrier Function by Microbial Metabolites. Cell. Mol. Gastroenterol. Hepatol..

[17] Aguanno D., Coquant G., Postal B.G., Osinski C., Wieckowski M., Stockholm D., Grill J.P., Carrière V., Seksik P., Thenet S. (2020). The intestinal quorum sensing 3-oxo-C12:2 Acyl homoserine lactone limits cytokine-induced tight junction disruption. Tissue Barriers.

[18] Coquant G., Aguanno D., Pham S., Grellier N., Thenet S., Carrière V., Grill J.P., Seksik P. (2021). Gossip in the gut: Quorum sensing, a new player in the host-microbiota interactions. World J. Gastroenterol..

[19] Ali A., Tan H., Kaiko G.E. (2020). Role of the Intestinal Epithelium and Its Interaction With the Microbiota in Food Allergy. Front. Immunol..

[20] Okumura R., Takeda K. (2017). Roles of intestinal epithelial cells in the maintenance of gut homeostasis. Exp. Mol. Med..

[21] Allaire J.M., Crowley S.M., Law H.T., Chang S.Y., Ko H.J., Vallance B.A. (2018). The Intestinal Epithelium: Central Coordinator of Mucosal Immunity. Trends Immunol..

[22] Karmakar S., Deng L., He X.C., Li L. (2020). Intestinal epithelial regeneration: Active versus reserve stem cells and plasticity mechanisms. Am. J. Physiol.-Gastrointest. Liver Physiol..

[23] Chanez-Paredes S.D., Abtahi S., Kuo W.T., Turner J.R. (2021). Differentiating Between Tight Junction-Dependent and Tight Junction-Independent Intestinal Barrier Loss In Vivo. Methods Mol. Biol..

[24] Peterson L.W., Artis D. (2014). Intestinal epithelial cells: Regulators of barrier function and immune homeostasis. Nat. Rev. Immunol..

[25] Zuo L., Kuo W.T., Turner J.R. (2020). Tight Junctions as Targets and Effectors of Mucosal Immune Homeostasis. Cell. Mol. Gastroenterol. Hepatol..

[26] Soderholm A.T., Pedicord V.A. (2019). Intestinal epithelial cells: At the interface of the microbiota and mucosal immunity. Immunology.

[27] Pardo-Camacho C., González-Castro A.M., Rodiño-Janeiro B.K., Pigrau M., Vicario M. (2018). Epithelial immunity: Priming defensive responses in the intestinal mucosa. Am. J. Physiol.-Gastrointest. Liver Physiol..

[28] Mörbe U.M., Jørgensen P.B., Fenton T.M., von Burg N., Riis L.B., Spencer J., Agace W.W. (2021). Human gut-associated lymphoid tissues (GALT); diversity, structure, and function. Mucosal Immunol..

[29] Bamias G., Rivera-Nieves J., Grisham M.B., Said H.M. (2018). Chapter 65—Recruitment of Inflammatory and Immune Cells in the Gut. Physiology of the Gastrointestinal Tract.

[30] Habtezion A., Nguyen L.P., Hadeiba H., Butcher E.C. (2016). Leukocyte Trafficking to the Small Intestine and Colon. Gastroenterology.

[31] Atarashi K., Tanoue T., Shima T., Imaoka A., Kuwahara T., Momose Y., Cheng G., Yamasaki S., Saito T., Ohba Y. (2011). Induction of colonic regulatory T cells by indigenous Clostridium species. Science.

[32] Coquant G., Aguanno D., Brot L., Belloir C., Delugeard J., Roger N., Pham H.P., Briand L., Moreau M., de Sordi L. (2022). 3-oxo-C12:2-HSL, quorum sensing molecule from human intestinal microbiota, inhibits pro-inflammatory pathways in immune cells via bitter taste receptors. Sci. Rep..

[33] Andrews C., McLean M.H., Durum S.K. (2018). Cytokine Tuning of Intestinal Epithelial Function. Front. Immunol..

[34] Friedrich M., Pohin M., Powrie F. (2019). Cytokine Networks in the Pathophysiology of Inflammatory Bowel Disease. Immunity.

[35] Rath E., Haller D. (2022). Intestinal epithelial cell metabolism at the interface of microbial dysbiosis and tissue injury. Mucosal Immunol..

[36] Carrière V., Rodolosse A., Lacasa M., Cambier D., Zweibaum A., Rousset M. (1998). Hypoxia and CYP1A1 induction-dependent regulation of proteins involved in glucose utilization in Caco-2 cells. Am. J. Physiol..

[37] Girer N.G., Tomlinson C.R., Elferink C.J. (2020). The Aryl Hydrocarbon Receptor in Energy Balance: The Road from Dioxin-Induced Wasting Syndrome to Combating Obesity with Ahr Ligands. Int. J. Mol. Sci..

[38] Bock K.W. (2021). Aryl hydrocarbon receptor (AHR), integrating energy metabolism and microbial or obesity-mediated inflammation. Biochem. Pharmacol..

[39] Parada Venegas D., De la Fuente M.K., Landskron G., González M.J., Quera R., Dijkstra G., Harmsen H.J.M., Faber K.N., Hermoso M.A. (2019). Short Chain Fatty Acids (SCFAs)-Mediated Gut Epithelial and Immune Regulation and Its Relevance for Inflammatory Bowel Diseases. Front. Immunol..

[40] Glover L.E., Lee J.S., Colgan S.P. (2016). Oxygen metabolism and barrier regulation in the intestinal mucosa. J. Clin. Investig..

[41] Lavelle A., Sokol H. (2020). Gut microbiota-derived metabolites as key actors in inflammatory bowel disease. Nat. Rev. Gastroenterol. Hepatol..

[42] Postal B.G., Ghezzal S., Aguanno D., André S., Garbin K., Genser L., Brot-Laroche E., Poitou C., Soula H., Leturque A. (2020). AhR activation defends gut barrier integrity against damage occurring in obesity. Mol. Metab..

[43] Ara T., Hashimoto D. (2021). Novel Insights Into the Mechanism of GVHD-Induced Tissue Damage. Front. Immunol..

[44] Jian Y., Zhang D., Liu M., Wang Y., Xu Z.X. (2021). The Impact of Gut Microbiota on Radiation-Induced Enteritis. Front. Cell Infect. Microbiol..

[45] Page M.J., Kell D.B., Pretorius E. (2022). The Role of Lipopolysaccharide-Induced Cell Signalling in Chronic Inflammation. Chronic Stress.

[46] Steimle A., Autenrieth I.B., Frick J.-S. (2016). Structure and function: Lipid A modifications in commensals and pathogens. Int. J. Med. Microbiol..

[47] Di Tommaso N., Gasbarrini A., Ponziani F.R. (2021). Intestinal Barrier in Human Health and Disease. Int. J. Environ. Res. Public Health.

[48] Genser L., Aguanno D., Soula H.A., Dong L., Trystram L., Assmann K., Salem J.E., Vaillant J.C., Oppert J.M., Laugerette F. (2018). Increased jejunal permeability in human obesity is revealed by a lipid challenge and is linked to inflammation and type 2 diabetes. J. Pathol..

[49] Thaiss C.A., Levy M., Grosheva I., Zheng D., Soffer E., Blacher E., Braverman S., Tengeler A.C., Barak O., Elazar M. (2018). Hyperglycemia drives intestinal barrier dysfunction and risk for enteric infection. Science.

[50] Martini E., Krug S.M., Siegmund B., Neurath M.F., Becker C. (2017). Mend Your Fences: The Epithelial Barrier and its Relationship With Mucosal Immunity in Inflammatory Bowel Disease. Cell. Mol. Gastroenterol. Hepatol..

[51] Irvine E.J., Marshall J.K. (2000). Increased intestinal permeability precedes the onset of Crohn’s disease in a subject with familial risk. Gastroenterology.

[52] Tilg H., Zmora N., Adolph T.E., Elinav E. (2020). The intestinal microbiota fuelling metabolic inflammation. Nat. Rev. Immunol..

[53] Petit C.S., Barreau F., Besnier L., Gandille P., Riveau B., Chateau D., Roy M., Berrebi D., Svrcek M., Cardot P. (2012). Requirement of cellular prion protein for intestinal barrier function and mislocalization in patients with inflammatory bowel disease. Gastroenterology.

[54] Su L., Shen L., Clayburgh D.R., Nalle S.C., Sullivan E.A., Meddings J.B., Abraham C., Turner J.R. (2009). Targeted epithelial tight junction dysfunction causes immune activation and contributes to development of experimental colitis. Gastroenterology.

[55] He W.Q., Wang J., Sheng J.Y., Zha J.M., Graham W.V., Turner J.R. (2020). Contributions of Myosin Light Chain Kinase to Regulation of Epithelial Paracellular Permeability and Mucosal Homeostasis. Int. J. Mol. Sci..

[56] Delgado M.E., Brunner T. (2019). The many faces of tumor necrosis factor signaling in the intestinal epithelium. Genes Immun..

[57] Khan S., Luck H., Winer S., Winer D.A. (2021). Emerging concepts in intestinal immune control of obesity-related metabolic disease. Nat. Commun..

[58] Ye D., Ma I., Ma T.Y. (2006). Molecular mechanism of tumor necrosis factor-alpha modulation of intestinal epithelial tight junction barrier. Am. J. Physiol. Gastrointest. Liver Physiol..

[59] Ruder B., Atreya R., Becker C. (2019). Tumour Necrosis Factor Alpha in Intestinal Homeostasis and Gut Related Diseases. Int. J. Mol. Sci..

[60] D’Haens G.R., van Deventer S. (2021). 25 years of anti-TNF treatment for inflammatory bowel disease: Lessons from the past and a look to the future. Gut.

[61] Iwaszkiewicz K.S., Schneider J.J., Hua S. (2013). Targeting peripheral opioid receptors to promote analgesic and anti-inflammatory actions. Front. Pharmacol..

[62] Thomas K.R., Watt J., Wu C.M.J., Akinrinoye A., Amjad S., Colvin L., Cowe R., Duncan S.H., Russell W.R., Forget P. (2022). Pain and Opioid-Induced Gut Microbial Dysbiosis. Biomedicines.

[63] Elbere I., Kalnina I., Silamikelis I., Konrade I., Zaharenko L., Sekace K., Radovica-Spalvina I., Fridmanis D., Gudra D., Pirags V. (2018). Association of metformin administration with gut microbiome dysbiosis in healthy volunteers. PLoS ONE.

[64] Rodriguez J., Hiel S., Delzenne N.M. (2018). Metformin: Old friend, new ways of action-implication of the gut microbiome?. Curr. Opin. Clin. Nutr. Metab. Care.

[65] Chen L., Wang J., You Q., He S., Meng Q., Gao J., Wu X., Shen Y., Sun Y., Wu X. (2018). Activating AMPK to Restore Tight Junction Assembly in Intestinal Epithelium and to Attenuate Experimental Colitis by Metformin. Front. Pharmacol..

[66] Wu W., Wang S., Liu Q., Shan T., Wang Y. (2018). Metformin Protects against LPS-Induced Intestinal Barrier Dysfunction by Activating AMPK Pathway. Mol. Pharm..

[67] Wu Z., Xu C., Zheng T., Li Q., Yang S., Shao J., Guan W., Zhang S. (2022). A critical role of AMP-activated protein kinase in regulating intestinal nutrient absorption, barrier function, and intestinal diseases. J. Cell. Physiol..

[68] Camilleri M. (2021). Human Intestinal Barrier: Effects of Stressors, Diet, Prebiotics, and Probiotics. Clin. Transl. Gastroenterol..

[69] Ghezzal S., Postal B.G., Quevrain E., Brot L., Seksik P., Leturque A., Thenet S., Carrière V. (2020). Palmitic acid damages gut epithelium integrity and initiates inflammatory cytokine production. Biochim. Biophys. Acta Mol. Cell Biol. Lipids.

[70] Suzuki T. (2020). Regulation of the intestinal barrier by nutrients: The role of tight junctions. Anim. Sci. J..

[71] Di Caro S., Fragkos K.C., Keetarut K., Koo H.F., Sebepos-Rogers G., Saravanapavan H., Barragry J., Rogers J., Mehta S.J., Rahman F. (2019). Enteral Nutrition in Adult Crohn’s Disease: Toward a Paradigm Shift. Nutrients.

[72] Mitrev N., Huang H., Hannah B., Kariyawasam V.C. (2021). Review of exclusive enteral therapy in adult Crohn’s disease. BMJ Open Gastroenterol..

[73] Ihara S., Hirata Y., Koike K. (2017). TGF-β in inflammatory bowel disease: A key regulator of immune cells, epithelium, and the intestinal microbiota. J. Gastroenterol..

[74] Karczewski J., Troost F.J., Konings I., Dekker J., Kleerebezem M., Brummer R.J., Wells J.M. (2010). Regulation of human epithelial tight junction proteins by Lactobacillus plantarum in vivo and protective effects on the epithelial barrier. Am. J. Physiol. Gastrointest. Liver Physiol..

[75] Rao R.K., Samak G. (2013). Protection and Restitution of Gut Barrier by Probiotics: Nutritional and Clinical Implications. Curr. Nutr. Food Sci..

[76] Yan J., Sheng L., Li H. (2021). Akkermansia muciniphila: Is it the Holy Grail for ameliorating metabolic diseases?. Gut Microbes.

[77] Bian X., Wu W., Yang L., Lv L., Wang Q., Li Y., Ye J., Fang D., Wu J., Jiang X. (2019). Administration of Akkermansia muciniphila Ameliorates Dextran Sulfate Sodium-Induced Ulcerative Colitis in Mice. Front. Microbiol..

[78] Plovier H., Everard A., Druart C., Depommier C., Van Hul M., Geurts L., Chilloux J., Ottman N., Duparc T., Lichtenstein L. (2017). A purified membrane protein from Akkermansia muciniphila or the pasteurized bacterium improves metabolism in obese and diabetic mice. Nat. Med..

[79] Van Nood E., Vrieze A., Nieuwdorp M., Fuentes S., Zoetendal E.G., de Vos W.M., Visser C.E., Kuijper E.J., Bartelsman J.F., Tijssen J.G. (2013). Duodenal infusion of donor feces for recurrent Clostridium difficile. N. Engl. J. Med..

[80] Baunwall S.M.D., Lee M.M., Eriksen M.K., Mullish B.H., Marchesi J.R., Dahlerup J.F., Hvas C.L. (2020). Faecal microbiota transplantation for recurrent Clostridioides difficile infection: An updated systematic review and meta-analysis. EClinicalMedicine.

[81] Ooijevaar R.E., Terveer E.M., Verspaget H.W., Kuijper E.J., Keller J.J. (2019). Clinical Application and Potential of Fecal Microbiota Transplantation. Annu. Rev. Med..

[82] Danne C., Rolhion N., Sokol H. (2021). Recipient factors in faecal microbiota transplantation: One stool does not fit all. Nat. Rev. Gastroenterol. Hepatol..

[83] Keller J.J., Ooijevaar R.E., Hvas C.L., Terveer E.M., Lieberknecht S.C., Högenauer C., Arkkila P., Sokol H., Gridnyev O., Mégraud F. (2021). A standardised model for stool banking for faecal microbiota transplantation: A consensus report from a multidisciplinary UEG working group. United Eur. Gastroenterol. J..

[84] Aguanno D., Metwaly A., Coleman O.I., Haller D. (2022). Modeling microbiota-associated human diseases: From minimal models to complex systems. Microbiome Res. Rep..

